# Resistance to Crayfish Plague: Assessing the Response of Native Iberian Populations of the White-Clawed Freshwater Crayfish

**DOI:** 10.3390/jof8040342

**Published:** 2022-03-25

**Authors:** María Martínez-Ríos, Sara Lapesa-Lázaro, Jokin Larumbe-Arricibita, Fernando Alonso-Gutiérrez, Francisco Javier Galindo-Parrila, Laura Martín-Torrijos, Javier Diéguez-Uribeondo

**Affiliations:** 1 Departamento de Micología, Real Jardín Botánico-CSIC, Plaza Murillo 2, 28014 Madrid, Spain; maria.mr@rjb.csic.es (M.M.-R.); lmtorrijos@rjb.csic.es (L.M.-T.); 2 Servicio Provincial de Teruel, Departamento de Desarrollo Rural y Sostenibilidad, Calle San Francisco 21, 44001 Teruel, Spain; slapesa@aragon.es; 3 Sección de Hábitats, Servicio de Conservación de la Biodiversidad, C/González Tablas, 9, 31005 Navarra, Spain; jlarumar@navarra.es; 4 Servicio de Medio Natural y Biodiversidad, Consejería de Desarrollo Sostenible, Delegación Provincial de Cuenca, Junta de Comunidades de Castilla-La Mancha, Calle Colón 2, 16194 Cuenca, Spain; falonso@jccm.es; 5 Evaluación y Planificación de Recursos Cinegéticos y Piscícolas, Gerencia de Granada, Calle Joaquina Eguaras 10, 18013 Granada, Spain; fgalindo@agenciamedioambienteyagua.es

**Keywords:** crayfish plague, *Austropotamobius*, resistance, mean survival time, cumulative mortality progress, immune response, melanization

## Abstract

Crayfish plague, caused by the oomycete pathogen *Aphanomyces astaci*, is one of the most devastating of the emerging infectious diseases. This disease is responsible for the decline of native European and Asian freshwater crayfish populations. Over the last few decades, some European crayfish populations were reported to display partial to total resistance to the disease. The immune response in these cases was similar to that exhibited by the natural carriers of the pathogen, North American freshwater crayfish, e.g., weak-to-strong melanization of colonizing hyphae. We tested the degree of resistance displayed by 29 native Iberian populations of *Austropotamobius pallipes* that were challenged by zoospores of the pathogen. We measured the following parameters: (i) mean survival time, (ii) cumulative mortality, and (iii) immune response, and found that the total cumulative mortality of all the challenged populations was 100%. The integration of the results from these parameters did not allow us to find differences in resistance towards *A. astaci* among the northern and central populations of the Iberian Peninsula. However, in the southern populations, we could identify four distinct population responses based on an evaluation of a GLM analysis. In the first case, the similar response could be explained by the effect of a pathogen strain with a lower-than-expected virulence, and/or an actual increase in resistance. In the Southern populations, these differences appear to be the consequence of either whole population or individual resistance. Individuals that survived for a longer period than the others showed a stronger immune response, i.e., presence of partially or fully melanized hyphae, which is similar to that of North American crayfish species. This might be the consequence of different mechanisms of resistance or/and tolerance towards *A. astaci*.

## 1. Introduction

Crayfish plague is a devastating emerging infectious disease in wildlife. This disease is caused by the pathogen *Aphanomyces astaci* and is responsible for the decline, and even extinction, of native European and Asian freshwater crayfish populations [1]. *Aphanomyces astaci* is an oomycete, which is a group of organisms that rely on biflagellate zoospores to spread and colonize hosts. This pathogen originated in North America [2,3] and is listed among the 100 World’s Worst Invasive Alien Species [4]. The immune system of North American crayfishes, the natural carriers of this pathogen [3,5], is in a balanced relationship with infections by *A. astaci*, which allows the host to coexist with the pathogen by controlling it as a chronic infection [6,7,8]. Despite having a strong response, hosts occasionally die of crayfish plague when the host–pathogen balance is disrupted by the effects of stressors [6,8,9,10,11].

The high resistance to crayfish plague in natural carriers is linked to the expression of prophenoloxidase (proPO) in the hemocytes of hosts [12]. This enzyme is the end component of the proPO-activating system, a cascade of serine proteinases [13,14] and proteins with the capacity to bind molecules characteristically present in microorganisms, such as β-1,3-glucans [15,16], lipopolysaccharides [17] or peptidoglycans [18,19]. During infection by *A. astaci*, hemocytes can encapsulate hyphae and produce melanin layers with fungistatic and fungitoxic properties [20], thus, preventing the pathogen colonization of the host. In contrast to the North American carriers, native European, Asian, South American, and Australasian crayfish species are highly susceptible to crayfish plague and die soon after infection [2,21,22,23]. In these species, hemocytes react very slowly to the growth of hyphae of *A. astaci* [5,7,12] and, due to this slow immune response, infected animals often present a 100% mortality rate. The pathogen kills the host and often all the individuals of a population within a few days [12,21,23].

Imports of North American freshwater crayfishes to Europe during the 19th and 20th centuries resulted in the introduction of *A. astaci* to the region and the subsequent crayfish plague outbreaks [24,25,26,27,28,29,30,31] and devastation up to more than 80% of the native freshwater crayfish populations [32]. In the last few years, cases of resistance against crayfish plague have been reported for some European populations. For instance, Makkonen et al. [33,34] reported increased resistance to infection by a less virulent haplotype (a-haplotype) of *A. astaci* under experimental conditions in some Finnish populations of the noble crayfish (*Astacus astacus*). In these studies, the authors observed partial resistance to the infection: partial melanization of hyphae, longer survival periods, and variability in the mortality rate of populations. More recently, Francesconi et al. [35] reported total resistance of one of these Finnish populations, i.e., Lake Rytky, when challenged with another strain of the a-haplotype.

In 2017, Martín-Torrijos et al. [36] tested the resistance of geographically isolated populations of the native white-clawed crayfish (*Austropotamobius pallipes*) from Pyrenean areas and found populations showing partial resistance, similar to that described for the Finnish populations of the noble crayfish [33,34,35]. More importantly, these authors identified the first European crayfish population to show 100% survival against infection by the most virulent strains of *A. astaci* (d1 and d2-haplotypes) known under laboratory conditions [23,37]. The immune response observed in individual crayfish showing total resistance was similar to that exhibited by the North American species: strong melanization and encapsulation of colonizing hyphae [36].

During the 1970s, two carriers, the signal crayfish (*Pacifastacus leniusculus*) and the red swamp crayfish (*Procambarus clarkii*), were introduced to the Iberian Peninsula, where *A. pallipes* is the only native freshwater crayfish species; soon after, the region experienced a series of mass mortalities of the native crayfish [26,31,38]. These events resulted in the extinction of the native species in Portugal and in several regions of Spain (e.g., Extremadura, Madrid, and Murcia) or a drastic decline in other areas of Spain, where the species has been listed as “vulnerable” or “at risk of extinction” [26,31,39,40]. The recent discovery of populations of *A. pallipes* in the Pyrenees showing increased resistance to crayfish plague raises the question of whether other populations and/or individuals are also resistant, and if so, to what extent.

Assessing the resistance of susceptible crayfish species to crayfish plague is complicated by the following reasons: (i) *A. astaci* spreads by biflagellate zoospores, the infective unit [41]. Zoospores possess a fragile plasmatic membrane that can be easily compromised by changes in chemical composition (e.g., pH) or physical conditions (e.g., temperature or agitation) [37], which can result in zoospore encystment and, therefore, a reduction in virulence [41]; (ii) the haplotypes of *A. astaci* vary in virulence. For example, a-haplotype has lower virulence than b-haplotype [33,34,35], but b, d1 and d2-haplotypes have a similar level of virulence [8]. The d1 and d2-haplotypes can also infect at a water temperature more than 5 °C higher than that of the other haplotypes [23,37]; (iii) pathogen strains can lose their sporulation capacity and virulence under stocking conditions [33,42]; (iv) the susceptibility of crayfishes may vary according to life stage [43] or maintenance conditions (Diéguez-Uribeondo personal observation); and (v) native European freshwater crayfish are endangered species, making the logistics and experimental design challenging, from obtaining the appropriate sampling and experimental permits to gathering a sufficient number of specimens per population to synchronize experiments.

In this study, we use two strains of the virulent d2-haplotype to assess the degree of resistance of other populations of *A. pallipes* in the Iberian Peninsula by measuring three key parameters of the hosts response: (i) mean survival time; (ii) cumulative mortality through assessing of the total cumulative mortality, the cumulative mortality every week and the cumulative mortality progress; and (iii) immune response. For this purpose, we performed a challenge experiment with a total of 29 populations from the Iberian Peninsula: 15 from central or northern regions and 14 from southern regions. These populations cover the majority of the genetic diversity of the species in the Iberian Peninsula [3].

## 2. Materials and Methods

### 2.1. Crayfish Sampling

**Figure 1:**
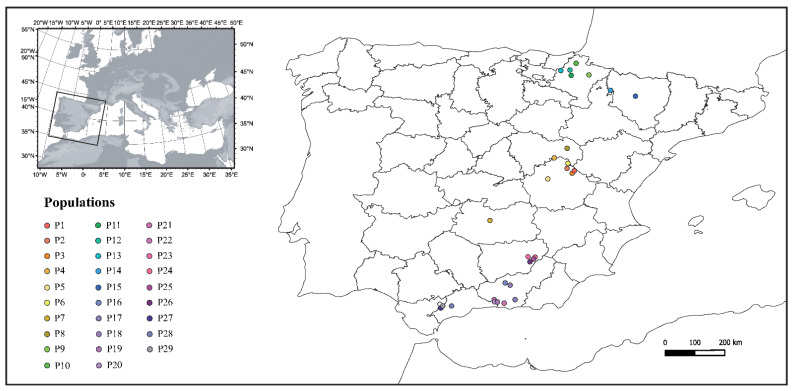
The location of the 29 selected populations of *Austropotamobius pallipes* in the Iberian Peninsula.

Specimens of the white-clawed crayfish *A*. *pallipes* were collected from 29 native populations located throughout the Iberian Peninsula, covering the genetic diversity of the species [3] and distribution in this region [44] (Figure 1, Table 1). None of the populations included in this study co-occur with any of the North American crayfish species. Due to the difficulty of synchronizing specimen collection, the populations were collected over two periods by region: the central and northern populations (P1–P15) were collected during the 2016–2017 season, and the southern ones (P16–P29) were collected during the 2020–2021 season. Individuals in each population were marked with a colored tag placed around the top of the abdomen prior to the population being placed in separate 0.80 m × 0.50 m × 0.40 m aquaria containing around 20 L of aerated filtered pond water and hiding places. The specimens were allowed to acclimatize for a week prior to any experimental manipulation. They were fed weekly with a diet of potatoes and carrots and monitored daily to remove any dead crayfish and excess debris as described by Martín-Torrijos et al. [36].

### 2.2. Challenge Experiments with Aphanomyces astaci

Due to the difference in the timing of sampling, we performed two independent challenge experiments: the first one (Experiment 1) was with the 15 central and northern populations collected in 2016–2017 (P1 to P15), and the second (Experiment 2), with the 14 southern populations collected in 2020–2021 (P16 to P29). For Experiment 1, we used the genome sequenced AP03 strain that belong to the d2-haplotype of *A*. *astaci* (GenBank accession number KX405004). For Experiment 2, we used the strain CCRJB_109 of the d2-haplotype, which has been recently isolated (GenBank accession numbers OK275539 and OK275540). Both strains were isolated from specimens of two populations of *P*. *clarkii* located in the surroundings of Garrotxa Natural Park (Gerona, Spain). They were obtained from the fungal collection of the Royal Botanical Garden-CSIC (Madrid, Spain). Zoospores were produced using the protocol described by Diéguez-Uribeondo et al. [37]. The number of crayfish samples from a population varied between 2 and 10, depending on how many we were able to find on the day of sampling. For this reason, the number of specimens analyzed differed in the two experiments (Table 1). Crayfish were placed and pooled into four experimental aquaria: replicate 1, 2, and 3, and control. Zoospores were added to only the replicate 1, 2, and 3 aquaria, and at a final concentration of 100 zoospores mL^−1^ ca per aquaria. No zoospores were added to the control aquarium. The experiment was followed for 60 days, and the temperature, oxygen levels, and water quality of the aquaria were checked daily. Water was replaced weekly to remove excess dirtiness. Dead individuals were quickly removed and processed for macroscopic and microscopic analyses before being preserved in 96% alcohol.

### 2.3. Mean Survival Time and Cumulative Mortality

After challenging the crayfish with the zoospores of *A*. *astaci*, we monitored their mortality over a period of 60 days. To evaluate the degree of resistance of the challenged populations, we measured the following variables: (i) the mean survival time, i.e., the mean number of days that crayfish survived from the start of the experiment; and (ii) the cumulative mortality through the total cumulative mortality, i.e., the proportion of challenged crayfish that died by the end of the experimental period, and cumulative mortality progress analyzing the mortality curves of the populations that showed significant differences in mean survival time. Moreover, we calculated the cumulative mortality every week per experiment as the percentage of cumulative mortality per week after pathogen exposure to identify peak periods of mortality.

We performed a Kaplan–Meier survival test (Log-Rank, Mantel-Cox) to evaluate differences in survival time among the controls and replicates. An ANOVA was performed to assess whether differences in mean survival time and in the total cumulative mortality among populations of each experiment were significant. If significant differences were found, we then performed a post hoc Tukey’s test to determine where the significant differences were. For assessing the cumulative mortality progress, we grouped the mortality data of the populations into different subsets according to the degrees of resistance reveled by the mean survival time. Each data subset was fitted to a generalized linear model (GLM) of proportional response with a binomial family of error distributions [45], using the R packages “car” [46]. For each GLM, we evaluated the residuals plots to analyze the adequacy of the model, using the R package “DHARMa” [47]. Moreover, we checked that there was no overdispersion, using the R package “aods3” [48], and, if not, it was corrected using a quasibinomial family of error distributions. Finally, we compared the Akaike information criterion (AIC) values derived from the GLMs obtained, using the R package “MuNIn” [49], and, so, we identified the model that best fit the data subset (i.e., the model with the minimum AIC value). All statistical analyses were performed in R v.3.4.1 [50].

### 2.4. Macroscopic and Microscopic Examination

All crayfish were examined before the start of the experiments to ensure they did not show any signs of previous colonization by the pathogen and resistance by checking for the presence of melanized hyphae. After being challenged with zoospores, crayfish were checked daily for disease symptoms. Dead crayfish were removed and examined, both macroscopically and microscopically, for the presence of melanized areas. For the microscopic examination, the sub-abdominal cuticle was removed and observed on an inverted microscope Olympus CKX41SF (Olympus Optical, Tokyo, Japan). Light micrographs were captured using a Qimaging Micropublisher 5.0 digital camera (Qimaging, Burnaby, BC, Canada). Digital image analysis was performed using the software Syncroscopy-Automontage (Microbiology International Inc., Frederick, MD, USA.) as described by Diéguez-Uribeondo et al. [51].

### 2.5. Molecular Analysis: Aphanomyces astaci-Specific PCR Test and rnnL and rnnS mtDNA Loci Sequencing

After the microscopic examination, all crayfish were tested for the presence of *A*. *astaci* after the challenge experiments using PCR-based analyses described by Oidtmann et al. [52]. All the crayfishes used in the challenge experiments were collected from populations that do not co-occur with any of the invasive freshwater crayfish species; therefore, they are not suspected to have been previously exposed to crayfish plague, also supported by the fact that none of the collected specimens showed any macroscopic signs of resistance prior to the experiment.

Genomic DNA was extracted from a piece of sub-abdominal cuticle using the E.Z.N.A. Insect DNA Kit (Omega bio-tek, Norcross/Atlanta, GA, USA). To test the presence of *A. astaci*, we performed a single round PCR using primers specific to the nrITS region of *A. astaci* according to the assay described by Oidtmann et al. [52]. The diagnostic primers used were 42 [52] and 640 [53], which are anchored in the ITS1 and ITS2 regions, respectively, and amplify the ITS1 and ITS2 surrounding the 5.8 S rDNA. Samples that tested positive for the pathogen’s presence were then analyzed to identify the intraspecific diversity of *A. astaci* using primers specific to the mitochondrial ribosomal small (rnnS) and large (rnnL) subunits [54]. Each round of PCR (nrITS, rnnS, and rnnL) was performed under the following cycling conditions: initial denaturation at 95 °C for 2 min; followed by 35 cycles at 95 °C for 30 s, 59 °C for 30 s, and 72 °C for 30 s; and a final extension step at 72 °C for 10 min. DNA extracted from pure cultures of strain AP03 and CCRJB_109 of the d2-haplotype were used as a positive control for Experiment 1 and 2, respectively. A negative control was also included. Three μL aliquots of the amplification product were analyzed by electrophoresis in 1% agarose TAE gels stained with SBYR1Safe (Thermo Fisher Scientific, Waltham, MA, USA). Double-stranded PCR products were sequenced using an automated sequencer (Applied Biosystems 3730xl DNA, Macrogen, The Netherlands). Sequences were assembled and edited using the program Geneious v6.14 8 [55], and a BLAST search was run on each sequence to confirm the strain found in the dead crayfish was the same used in the zoospore challenge, thus complying with Koch’s postulates.

## 3. Results

### 3.1. Aphanomyces astaci-Challenge Experiments

**Figure 2:**
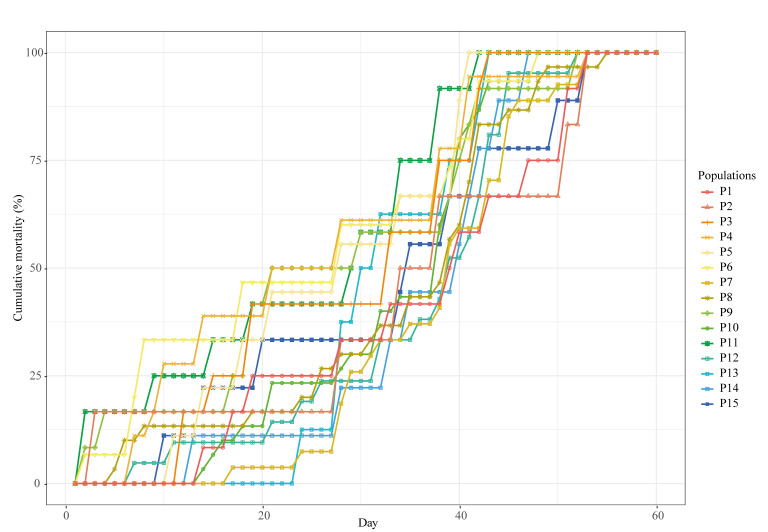
Cumulative mortality progress of the populations of *Austropotamobius pallipes* exposed to *Aphanomyces astaci* zoospores from Experiment 1. The challenged experiment was performed with 15 native Iberian crayfish populations from central and northern regions. Populations were monitored for 60 days after exposure to zoospores. Crayfishes of the control died on days 6, 7, 9, 12, 14, 17, 18, 28, 29, 40, 41, and 45 after *Aphanomyces astaci* zoospores challenge. The control is not represented in the figure.

**Figure 3:**
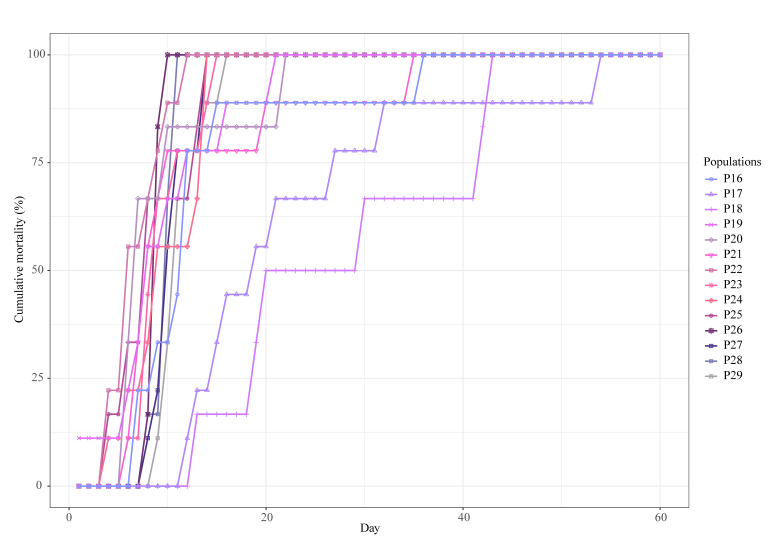
Cumulative mortality progress of the populations of *Austropotamobius pallipes* exposed to *Aphanomyces astaci* zoospores from Experiment 2. The challenged experiment was performed with 14 native Iberian crayfish populations from southern regions. Populations were monitored for 60 days after exposure to zoospores. Crayfishes of the control died on days 3, 10, 13, 14, 23, 28, 39, and 41 after *Aphanomyces astaci* zoospores challenge. The control is not represented in the figure.

By the end of the experimental period (60 days), all *A. astaci*-challenged crayfish had died. In general, infected crayfish by this pathogen start dying from day 6 after *A. astaci* zoospore challenge [2,7,21,23,56]. The majority died after the first week, supporting the findings of previous studies. Our macroscopic and microscopic examinations and molecular analysis of those that died in the first five days of the experiment revealed that they did not die of crayfish plague. Therefore, we excluded the crayfish that died prior to day six of the experiment from the statistical analyses. The overall mortality period of both experiments was nearly the same: for Experiment 1, it was from day 6 to day 55 (Figure 2), and for Experiment 2, from day 6 to day 54 (Figure 3). Regarding the control, 17 out of the 51 individuals in Experiment 1 died during the 60 days experimental period and, in Experiment 2, 8 out of the 35 individuals died. 

### 3.2. Mean Survival Time and Cumulative Mortality

By 60 days post-exposure, all the crayfish from the challenged populations had died, making their total cumulative mortality 100% (Table 2). The total mortality cumulative in controls of the Experiment 1 and Experiment 2 was 33.33% and 22.86%, respectively. The ANOVA showed differences in the total cumulative mortality between the controls and replicates, which were statistically significant for Experiment 1 (F-statistic: 147.2 on 3 and 271 degrees of freedom, *p*-value: < 2.2 × 10^−16^) and Experiment 2 (F-statistic: 132.4 on 3 and 136 degrees of freedom, *p*-value: < 2.2 × 10^−16^), but those between replicates and populations were not. According to the Kaplan–Meier survival test (Log-Rank, Mantel-Cox), differences in the survival time between the controls and replicates were statistically significant (χ^2^ = 161 on 3 degrees of freedom, *p*-value = 2 × 10^−16^), but those between replicates were not (χ^2^ = 4.4 on 2 degrees of freedom, *p*-value = 0.1). Given this, we combined the data from the three replicates of each population in the two experiments. The ANOVA for Experiment 1 showed there were no statistically significant differences in mean survival time between populations (F-statistic: 1.65 on 14 and 209 degrees of freedom, *p*-value: 0.06834). Population P7 had the highest mean survival time (38 days sd ± 9.05), and population P11, the lowest (24.33 days sd ± 14.38) (Table 2). By contrast, the ANOVA for Experiment 2 showed statistically significant differences in mean survival time between populations (F-statistic: 5.031 on 13 and 94 degrees of freedom, *p*-value: 1.154 × 10^−6^). Populations P17 and P18 had the two highest mean survival times (in days, 23.22 sd ± 13.28 and 24.00 sd ± 11.73, respectively), and population P22 had the lowest (7.22 days sd ± 2.73) (Table 2). A post hoc Tukey’s test confirmed that the differences in mean survival time between these two populations (P17 and P18) and the other southern populations were statistically significant (except between P17 and P21), while the difference between them was not (Table 3).

We also compared cumulative mortality by week and observed that, in Experiment 2, the major mortality event occurred in the second week after exposure to zoospores (Table 4). During this period, around 64% of the crayfish died. From the third week to the end of the experimental period, only isolated deaths were observed. In Experiment 1, the mortality events were more evenly distributed and later compared with Experiment 2, with the highest weekly mortality, approximately 32%, occurring during the fifth week (Table 4).

Since we obtained significant differences in the mean survival time Experiment 2, we statistically analyzed the cumulative mortality progress of the southern populations. Using data derived from the mean survival times and the cumulative mortality curves shown in Figure 3, we grouped the cumulative mortality data of these populations into three data sets according to the degrees of resistance. The first (data set 1) consisted of the data of two resistance groups (populations P17 and P18 together and all the other populations); the second (data set 2) comprised three resistance groups (population P17, population P18, and the other populations); and, finally, the third (data set 3) was formed of four resistance groups (population P17, population P18, populations P16 and P21 together, and the rest of the populations). We compared the AIC values of each fitted GLM (Table 5) and we obtained that the data set 3 model, comprising four resistance groups, was the best fit model to the data subsets, i.e., the model with the minimum AIC value.

### 3.3. Macroscopic and Microscopic Examinations

**Figure 4:**
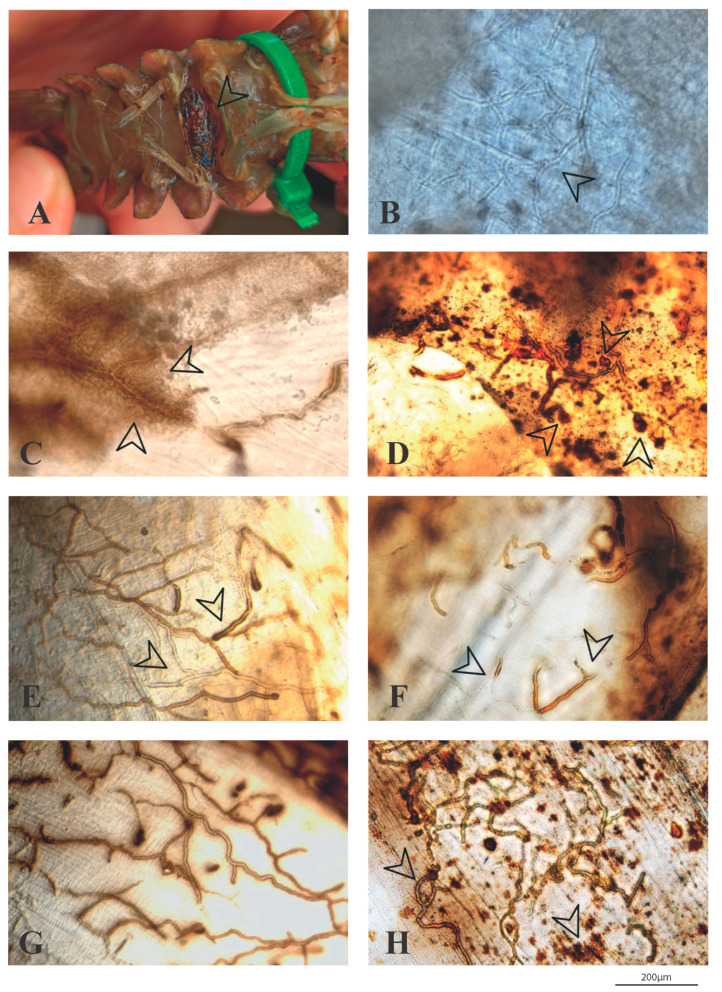
Immune reaction of *Austropotamobius pallipes* after being challenged with *Aphanomyces astaci*. (**A**) Macroscopic signs of infection in the form of a melanized patch in the abdominal cuticle of crayfish showing resistance against *A. astaci*. Abdominal cuticle of susceptible crayfish that died after being inoculated with zoospores of *A. astaci*: (**B**) growth of non-melanized hyphae; (**C**) aggregation of haemocytes encapsulating hyphae of *A. astaci* with melanin molecules secreted; (**D**) arrows mark the starting points of the colonization process, which is surrounded by melanin; (**E**) melanized (right arrow) and non-melanized hyphae (left arrow); (**F**) partially melanized hyphae; (**G**,**H**) images showing a general view of an infected cuticle with strongly melanized hyphae and melanin deposits (arrow). Bar = 200 μm.

The macroscopic examination of the challenged individuals revealed the majority of individuals did not show any visible signs of resistance and only some had melanized spots on the cuticle (Figure 4A). The microscopic examination of dead crayfish showed the presence of abundant non-melanized hyphae growing within the sub-abdominal cuticle. These hyphae had morphological characteristics typical of *A*. *astaci* (rounded tips and around 10 μm in diameter) (Figure 4B). We also observed crayfish with partially and complete melanized hyphae (i.e., signs of resistance against *A. astaci)* in all the populations (Figure 4C–H), with some individuals showing aggregation of haemocytes encapsulating the hyphae (Figure 4C). Generally, specimens that that died at later stages (e.g., some crayfish of P16 and P21) or started to die later than individuals from other populations (i.e., individuals from P17 and P18) of the experiments showed a greater abundance of melanized hyphae, whereas those that died at earlier stages scarcely had any melanized hyphae.

### 3.4. Molecular Analyses

All the crayfish exposed to zoospores of *A*. *astaci* tested positive for the presence of the pathogen, except for those that died within the first five days after exposure. All the controls tested negative. The DNA sequences of the pathogen isolated from positive crayfish were identical to the isolate of *A*. *astaci* used for each challenge experiment. That is, sequences of *A. astaci* isolated from the first and the second experiment showed 100% similarity to the AP03 and the CCRJB_109 isolate, respectively.

## 4. Discussion

In this study, we identified differences in the degree of resistance towards *A. astaci* among Iberian populations of *A. pallipes*, at both the population and the individual level. These populations differed in mean survival time and cumulative mortality progress after exposure to the zoospores of *A. astaci*. In particular, the southern populations P17 and P18 showed a significant difference in these two parameters compared with the other southern populations. However, under our experimental conditions, we did not observe any population or individual that showed signs of total resistance similar to those previously described for populations in the Pyrenees [36].

As judged by the results, the increased resistance observed in populations P17 and P18 seems to be the consequence of a more efficient immune response. The microscopic observation of the individuals of these two populations revealed melanization and encapsulation of hyphae, signs of a strong immune response. These signs were also seen in some individuals from other populations, mainly P16 and P21, that survived the infection for longer than other individuals of the same population. Our observations corroborate previous studies that have shown that an increase in melanization can stop the progression of an infection by *A. astaci* [20,36]. Correlations between degree of melanization and resistance towards a disease have been reported in other invertebrates. For instance, in the cricket *Allonemobius socius*, individuals with a high degree of melanization in their cuticles also have greater PO activity and resistance to the bacteria *Serratia marcescens* [57]. Water striders (*Aquarius naja*) that survive overwintering show increased melanization and an enhanced melanin-based immune defense against a nylon monofilament acting as a novel pathogen [58]. The pattern of immune responses observed in susceptible native freshwater crayfish species suggest that the mechanism of resistance may be present across populations, but also in isolated individuals. Our results were consistent with previous research showing that host resistance is based on the efficiency of the immune system to produce melanin, which slows the process of pathogen colonization and infection [13,59].

The pattern of cumulative mortality progress of the less resistant southern populations followed a typical sigmoidal (S-shaped) curve with an initial lag phase, followed by an exponential and then decline phase [60]. In contrast, the populations with increased resistance showed a “staggered”-shaped curve, a consequence of delays in mortality and secondary infections. The delays observed since the early stages of infection in populations P17 and P18 and at late stages in populations P16 and P21 seem to be a consequence of a more efficient immune response (based on increased melanization in the infected cuticles) by the host. Secondary infections are caused by newly formed infective zoospores produced from previously infected crayfish, which release them while still alive [41,61,62]. Therefore, although infected crayfish were removed soon after death, they likely released new zoospores, causing the secondary infections.

The analysis of the mean survival allowed us to identify two populations that responded differently to *A. astaci*. However, when we analyzed the cumulative mortality progress caused by the pathogen, the response of the populations could be separated in four groups, which responded differently to the infection. These were the following organized from the highest to lowest degree of resistance: (i) the population P17, (ii) the population P18, (iii) populations P16 and P21, and (iv) the rest of the populations. This analysis allowed us to have an overall picture of the disease progress of the different resistance groups, which integrates results from mean survival time and defense response. Thus, we found that crayfish from populations P17 and P18 were the most resistant groups and they behaved similarly. Their cumulative mortality progress curve and mean survival time showed that they started to succumb to infection several days later than the other populations as a result of a higher immune defense (i.e., melanin formation). Therefore, their mortality progress curves are delayed in time indicating an increase in resistance at a population level. Furthermore, some of the crayfish in populations 16 and 21 were able to resist more time to infection than their mean population. As a consequence, their cumulative mortality curves remained longer at the final stage than those of the other southern susceptible populations, revealing an increase in individual resistance.

No significant differences in crayfish plague resistance were observed in Experiment 1 with the central and northern populations. The cumulative mortality progress curves of the northern and central populations followed a “staggered” shape similar to those of the resistant southern populations. In this case, the response could be explained by the effect of a pathogen strain with a lower-than-expected virulence and/or an actual increase in resistance. Regarding the virulence, the strain used this experiment (AP03) was isolated long before (in 2006) the one used in Experiment 2 with the southern populations (CCRJB_109, which was isolated in 2021). Oomycetes can lose viability and virulence over time, particularly, *A. astaci* [33,42]. We suspect that loss of virulence likely explains the “staggered”-shaped mortality curves for these populations. However, for some of these populations, increased resistance may also be considered. For instance, the population P10 from Navarra was previously described as having increased resistance to *A. astaci* compared to other populations from the Pyrenees [36]. We speculate that the populations tested probably displayed the same level of resistance following exposure to a less virulent strain.

Overall, in this study, we showed that up to four Iberian populations of *A. pallipes* have higher resistance towards *A. astaci* and that differences can be also in some individuals from other populations. The immune response observed crayfish showing increase resistance at the individual and the population level is similar to that of North American crayfish species. We observed macroscopic melanized body spots and partially or completely melanized hyphae in the cuticles. These features have been associated with longer survival times and a slower mortality progress [33,34,36]. Immunological studies highlight that the mechanisms that the host defense system triggered to deal with a pathogen are different in terms of resistance and tolerance [63]. Thus, resistance refers to the ability to limit parasite burden, i.e., the ability to prevent pathogen entry and reduce their burden inside the host, while tolerance indicates the ability to limit the disease severity induced by a given parasite burden, i.e., the reaction of the host against the infection intensity [63,64]. In this study, we cannot formally distinguish between resistance and tolerance. However, assessing the disease severity among crayfish populations using different pathogen loads (zoospore concentrations) will be required in further studies. The results presented in this work set the basis for further studying whether infection severity between these populations could be the result of differences in tolerance rather than resistance in strict terms, and thus better understand the crayfish defense mechanism involved to control this disease. Moreover, the finding that native Iberian crayfish populations of *A. pallipes* can have different response towards *A. astaci* at both the population and individual level is of key importance for the conservation of this endangered species. Specimens from these populations can be selected for stocking and breeding programs with a careful planning and knowing also their genetic background to increase their resistance/tolerance or both to the crayfish plague pathogen.

## Figures and Tables

**Table 1:** Populations of *Austropotamobius pallipes* analyzed in the challenge experiments. For each population, the location and number of individuals per replicate and control and the total number used in these experiments are also indicated.

Population Name	Location	Experiment	Replicate 1	Replicate 2	Replicate 3	Control	Total Individuals
P1	Cuenca, Castilla-La Mancha	1	4	4	4	4	16
P2	Cuenca, Castilla-La Mancha	1	2	2	2	2	8
P3	Cuenca, Castilla-La Mancha	1	4	4	4	4	16
P4	Cuenca, Castilla-La Mancha	1	6	6	6	6	24
P5	Cuenca, Castilla-La Mancha	1	3	3	3	3	12
P6	Cuenca, Castilla-La Mancha	1	5	5	5	5	20
P7	Guadalajara, Castilla-La Mancha	1	9	9	9	4	31
P8	Ciudad Real, Castilla-La Mancha	1	10	10	10	7	37
P9	Navarra	1	4	4	4	2	14
P10	Navarra	1	10	10	10	3	33
P11	Navarra	1	4	4	4	2	14
P12	Navarra	1	7	7	7	4	25
P13	Navarra	1	3	3	3	2	11
P14	Huesca, Aragón	1	3	3	3	2	11
P15	Huesca, Aragón	1	3	3	3	1	10
P16	Granada, Andalucía	2	3	3	3	3	12
P17	Granada, Andalucía	2	3	3	3	3	12
P18	Granada, Andalucía	2	2	2	2	1	7
P19	Granada, Andalucía	2	3	3	3	3	12
P20	Granada, Andalucía	2	2	2	2	2	8
P21	Granada, Andalucía	2	3	3	3	3	12
P22	Jaén, Andalucía	2	3	3	3	3	12
P23	Jaén, Andalucía	2	3	3	3	3	12
P24	Jaén, Andalucía	2	3	3	3	3	12
P25	Jaén, Andalucía	2	3	3	3	2	11
P26	Jaén, Andalucía	2	3	3	3	1	10
P27	Málaga, Andalucía	2	3	3	3	3	12
P28	Málaga, Andalucía	2	2	2	2	2	8
P29	Málaga, Andalucía	2	2	2	2	3	9

**Table 2:** Total cumulative mortality and mean survival time of the challenged populations of *Austropotamobius pallipes*.

Population Name	Experiment	Total Cumulative Mortality	Mean Survival Time (Standard Deviation) (Days)
P1	1	100%	36.25 (sd ± 13.95)
P2	1	100%	34.33 (sd ± 16.46)
P3	1	100%	28.83 (sd ± 12.44)
P4	1	100%	25.50 (sd ± 14.75)
P5	1	100%	27.44 (sd ± 11.85)
P6	1	100%	24.60 (sd ± 15.76)
P7	1	100%	38.00 (sd ± 9.05)
P8	1	100%	33.7 (sd ± 13.46)
P9	1	100%	27.17 (sd ± 15.91)
P10	1	100%	33.53 (sd ± 9.92)
P11	1	100%	24.33 (sd ± 14.38)
P12	1	100%	35.52 (sd ± 11.68)
P13	1	100%	33.25 (sd ± 7.15)
P14	1	100%	35.89 (sd ± 10.40)
P15	1	100%	33 (sd ± 15.29)
Control	1	10.9%	47.84 (sd ± 19.05)
P16	2	100%	13.44 (sd ± 8.85)
P17	2	100%	23.22 (sd ± 13.28)
P18	2	100%	24.00 (sd ± 11.73)
P19	2	100%	9.89 (sd ± 5.86)
P20	2	100%	20.50 (sd ± 9.99)
P21	2	100%	12.22 (sd ± 9.51)
P22	2	100%	7.22 (sd ± 2.73)
P23	2	100%	9.78 (sd ± 2.99)
P24	2	100%	10.11 (sd ± 3.79)
P25	2	100%	13.33 (sd ± 3.14)
P26	2	100%	13.83 (sd ± 0.97)
P27	2	100%	10.78 (sd ± 2.05)
P28	2	100%	15.50 (sd ± 1.00)
P29	2	100%	11.56 (sd ± 2.19)
Control	2	7.89%	51.17 (sd ± 17.60)

**Table 3:** *p*-value of the Tukey’s test for the mean survival time between populations from Experiment 2. Statistically significant differences between populations are indicated in bold.

*p*-Value	P16	P17	P18	P19	P20	P21	P22	P23	P24	P25	P26	P27	P28	P29
P16														
P17	>0.05													
P18	**0.0084433**	>0.05												
P19	>0.05	**0.0051506**	**0.0002135**											
P20	>0.05	**0.0180352**	**0.0009389**	>0.05										
P21	>0.05	0.0516023	**0.0025667**	>0.05	>0.05									
P22	>0.05	**0.0006579**	**0.0000276**	>0.05	>0.05	>0.05								
P23	>0.05	**0.0045652**	**0.0001885**	>0.05	>0.05	>0.05	>0.05							
P24	>0.05	**0.0208085**	**0.0009735**	>0.05	>0.05	>0.05	>0.05	>0.05						
P25	>0.05	**0.0379016**	**0.0023556**	>0.05	>0.05	>0.05	>0.05	>0.05	>0.05					
P26	>0.05	**0.0098631**	**0.0004890**	>0.05	>0.05	>0.05	>0.05	>0.05	>0.05	>0.05				
P27	>0.05	**0.0130939**	**0.0005671**	>0.05	>0.05	>0.05	>0.05	>0.05	>0.05	>0.05	>0.05			
P28	>0.05	**0.0241095**	**0.0012926**	>0.05	>0.05	>0.05	>0.05	>0.05	>0.05	>0.05	>0.05	>0.05		
P29	>0.05	**0.0280708**	**0.0012905**	>0.05	>0.05	>0.05	>0.05	>0.05	>0.05	>0.05	>0.05	>0.05	>0.05	

**Table 4:** Cumulative mortality (%) of the *Aphanomyces astaci* challenged populations of *Austropotamobius pallipes* by week.

	Cumulative Mortality (%)	
Week	Experiment 1	Experiment 2
1	3.14	16.04
2	8.52	64.15
3	12.1	11.32
4	9.87	1.89
5	14.8	2.83
6	32.29	1.89
7	13	0.94
8	6.28	0.94
9	0	0

**Table 5:** AIC values of the comparison of GLMs fitted to data set 1, 2, and 3, based on the cumulative mortality of the southern populations of *Austropotamobius pallipes*. (See text for data set groupings).

GLM	AIC Value
Data set 1	2270.599
Data set 2	2261.121
Data set 3	2256.364

## Data Availability

All data supporting the findings of this study are presented within this article. Sequences AP03 and CCRJB_109 are deposited in GenBank.
